# The Place of Arsenic in the Production of Disease

**Published:** 1902-11-01

**Authors:** 


### Hospital Clinics and Medical Progress.

### THE PLACE OF ARSENIC IN THE PRODUCTION OF DISEASE.

For long it has been known that arsenic was capable under certain conditions of producing serious lesions. The very serious outbreak of arsenical poisoning arising from gross contamination of beer which recently caused so much suffering in the North of England,[1] has done much to arouse interest in the nature of the toxic action of arsenic. It is to be hoped that the Royal Commission, which is supposed to be collecting evidence regarding the presence of arsenic in food products, will soon place their report before us. Meanwhile, much important material has been collected and numerous suggestions have opened up fresh paths for investigation. It would seem that much of the "alcoholic neuritis" so long a common clinical entity in Manchester and district was really of arsenical origin. It is of course irrational and unscientific to jump to the conclusion, as some have done, that all peripheral neuritis in alcoholics was of arsenical causation; but the proof is clear that in some districts arsenic has for many years been present in appreciable quantities in malt liquors. There is also good ground for believing that the musculur degeneration which so conspicuously involved the myocardium of beer drinkers, producing the so-called "alcoholic dilation of the heart," formerly exceedingly common in Lancashire, also arose in many cases from the action of arsenic. It would seem that alcohol has the power of increasing the toxic action of arsenic on human tissues. An attempt has been made to show that beriberi, an important tropical disease fortunately but rarely met with in this country, is also dependent on the introduction of arsenic. The main evidence presented is that arsenic has been found in the hair of beriberi patients. But Dr. Patrick Manson has recently shown that Chinese tobacco often contains appreciable quantities of arsenic, introduced apparently mainly with the idea of giving the weed a garlic flavour. And Gautier, it will be remembered, has contended that arsenic must be considered a normal constituent of hair and other human tissues, although it must be admitted that such contention has not received adequate substantiation. Mr. Jonathan Hutchinson and others have shown that the keratosis produced by arsenic may pass on into a distinct epitheliomatous condition. But Mr. Hutchinson has recently gone much further and seeks to show that arsenic may have an ætiological relationship to cancer. In the October number of *The Polyclinic* he accepts as proved that chimney-sweepers' cancer is caused by the local irritation of arsenic. "The fact that neither coal-dust nor wood-ashes could cause it has long been recognised. Neither charcoal burners nor coal miners are liable to soot cancer. Its subjects are chimney - sweepers, stokers, gardeners, and others who come in contact with coal-soot. Now coal-soot contains large quantities of arsenic, and the condition of skin which it produces in chimney-sweepers, is in all respects, exactly like that which occurs occasionally in connection with the too long continued medicinal use of arsenic. The chain of evidence appears, therefore, to be complete." Mr. Hutchinson now advances still a step further: "That the habit of smoking is productive of cancer may be taken to be as well established as that exposure to coal-soot can cause it, but we have not as yet demonstrated that in this instance it is the presence of arsenic in tobacco that is influential." Mr. Hutchinson is rich in suggestions, but we venture to think that, until more adequate evidence is forthcoming, pathologists must abstain from any dogmatic expression which can only hinder progress and give rise to needless anxiety to the public. Mr. Hutchinson's contentions should receive respectful consideration, and must be submitted to impartial and thorough investigation. In seeking to unveil the mystery which for ages has surrounded the subject of malignant disease preconceived opinions, prejudice, and bias can have no part, but all evidence must be judicially weighed and scientifically sounded.

[1] *Medical Annual*, 1902, and "Arsenical Poisoning in Beer Drinkers," by T. N. Kelynack, M.D., and William Kirkby, F.L.S.

